# Structural insight into the hydrolase and synthase activities of an alkaline α-galactosidase from *Arabidopsis* from complexes with substrate/product

**DOI:** 10.1107/S2059798323000037

**Published:** 2023-01-20

**Authors:** Phimonphan Chuankhayan, Ruey-Hua Lee, Hong-Hsiang Guan, Chein-Chih Lin, Nai-Chi Chen, Yen-Chieh Huang, Masato Yoshimura, Atsushi Nakagawa, Chun-Jung Chen

**Affiliations:** a Life Science Group, Scientific Research Division, National Synchrotron Radiation Research Cente, Hsinchu 30076, Taiwan; b Institute of Tropical Plant Sciences and Microbiology, National Cheng Kung University, Tainan City 701, Taiwan; c Institute for Protein Research, Osaka University, Suita, Osaka 565-0871, Japan; d Department of Biotechnology and Bioindustry Sciences, National Cheng Kung University, Tainan City 701, Taiwan; e Department of Physics, National Tsing Hua University, Hsinchu 30013, Taiwan; f Department of Biological Science and Technology, National Chiao Tung University, Hsinchu 30010, Taiwan; Osaka University, Japan

**Keywords:** *Arabidopsis thaliana*, alkaline α-galactosidases, raffinose, RFO synthases, transferase mechanism

## Abstract

The alkaline α-galactosidase AtAkαGal3 from *Arabidopsis thaliana* catalyzes the hydrolysis of α-d-galactose from galacto-oligosaccharides under alkaline conditions. A phylogenetic analysis based on sequence alignment classifies AtAkαGal3 as more closely related to the raffinose family of oligosaccharide (RFO) synthases than to the acidic α-galactosidases. Here, thin-layer chromatography is used to demonstrate that AtAkαGal3 exhibits a dual function and is capable of synthesizing stachyose using raffinose, instead of galactinol, as the galactose donor. Crystal structures of complexes of AtAkαGal3 and its D383A mutant with various substrates and products, including galactose, galactinol, raffinose, stachyose and sucrose, are reported as the first representative structures of an alkaline α-galactosidase. The structure of AtAkαGal3 comprises three domains: an N-terminal domain with 13 antiparallel β-strands, a catalytic domain with an (α/β)_8_-barrel fold and a C-terminal domain composed of β-sheets that form two Greek-key motifs. The WW box of the N-terminal domain, which comprises the conserved residues FRSK_75_XW_77_W_78_ in the RFO synthases, contributes Trp77 and Trp78 to the +1 subsite to contribute to the substrate-binding ability together with the (α/β)_8_ barrel of the catalytic domain. The C-terminal domain is presumably involved in structural stability. Structures of the D383A mutant in complex with various substrates and products, especially the natural substrate/product stachyose, reveal four complete subsites (–1 to +3) at the catalytic site. A functional loop (residues 329–352) that exists in the alkaline α-galactosidase AtAkαGal3 and possibly in RFO synthases, but not in acidic α-galactosidases, stabilizes the stachyose at the +2 and +3 subsites and extends the catalytic pocket for the transferase mechanism. Considering the similarities in amino-acid sequence, catalytic domain and activity between alkaline α-galactosidases and RFO synthases, the structure of AtAkαGal3 might also serve a model for the study of RFO synthases, structures of which are lacking.

## Abstract 2

The alkaline α-galactosidase AtAkαGal3 from *Arabidopsis* exhibits a dual function where it can synthesize stachyose using raffinose, instead of galactinol, as the galactose donor. Crystal structures of complexes of AtAkαGal3 and its D383A mutant with various substrates and products, including galactose, galactinol, raffinose, stachyose and sucrose, reveal four complete subsites (–1 to +3) and a new secondary product-binding site, providing the first representative structure of an alkaline α-galactosidase and a model for the raffinose family of oligosaccharide synthases.

## 1. Introduction

α-Galactosidases (α-d-galactoside galactohydrolases; EC 3.2.1.22), which are widely found in microorganisms, plants, animals and humans, catalyze the removal of the nonreducing α-d-galactose moiety from galacto-oligosacharides, polysaccharides, galactolipids and glycoproteins (Henrissat *et al.*, 2001 ▸). Fabry disease is a rare genetic disease in humans that is due to a deficiency of α-galactosidase A, which is needed to catabolize the α-d-galactosyl moiety of sphingolipids in the lysosome (Calhoun *et al.*, 1985 ▸; Weidemann *et al.*, 2003 ▸). The hydrolytic specificity of α-galactosidases enables their application in the food industry to remove raffinose and to increase the yield of sucrose in the sugar beet industry (Shibuya *et al.*, 1995 ▸), to improve the gelling properties of galactomannans for use as food thickeners and to degrade the raffinose family of oligosaccharides (RFOs) in food and feed materials and in enzymotherapy (Guimarães *et al.*, 2001 ▸).

Based on their glycoside hydrolase (GH) signatures, α-galactosidases and RFO synthases belong to GH families 27 and 36, which are characterized by two conserved motifs: K*x*D and R*xxx*D (Henrissat & Romeu, 1995 ▸; Henrissat, 1991 ▸; Carmi *et al.*, 2003 ▸; Lee *et al.*, 2004 ▸; http://www.cazy.org/). Plant α-galactosidases can be further divided into acidic and alkaline forms, according to their optimal pH value for enzymatic activity. Alkaline α-galactosidases have optimal activities at neutral–alkaline pH values, whereas acidic α-galactosidases have optimal pH values of 3–6.5 (Lee *et al.*, 2004 ▸). Another major difference between these two types of α-galactosidase is that the total number of amino acids in alkaline α-galactosid­ases is about twice that in acidic α-galactosidases. A phylogenic analysis showed that the amino-acid sequences of alkaline α-galactosidases share a higher similarity to those of RFO synthases than to those of acidic α-galactosidases (Lee *et al.*, 2004 ▸). Both alkaline α-galactosidases and RFO synthases are unique to plants. Most studies have focused on acidic α-galactosidases (Keller & Pharr, 1996 ▸; Dey & Pridham, 1972 ▸; Herman & Shannon, 1985 ▸). Acidic α-galactosidases are involved in the mobilization of seed reserves of RFOs and the degradation of cell-wall galactomannan during seed germination (Corchete & Guerra, 1987 ▸). It has been suggested that these enzymes also play a role in cell-wall modification during fruit ripening (Kang & Lee, 2001 ▸; Nunan *et al.*, 2001 ▸).

The first alkaline α-galactosidase was uncovered in the Cucurbitaceae family (Gaudreault & Webb, 1986 ▸; Gao & Schaffer, 1999 ▸). High alkaline α-galactosidase activity was mostly found in roots and immature leaves in the early development stage; the activity declined with increasing leaf maturity in *Cucurbita pepo* (Gaudreault & Webb, 1986 ▸). High activity in an immature organ is due to the translocation of RFOs, the major galactosyl-sucrose oligosaccharides, from their source organs to developing organs such as leaves (Gaudreault & Webb, 1986 ▸) and fruits (Gao & Schaffer, 1999 ▸). Alkaline α-galactosidases can be found in more than one form in the Cucurbitaceae family, with varied substrate specificities. Alkaline α-galactosidases from *Cucurbita pepo*, *Cucumis sativus* and *Cucurbita maxima* prefer stachyose to raffinose, whereas in *Cucumis melo* fruit alkaline α-galactosidases of forms I and II exhibit a higher activity towards raffinose and stachyose, respectively (Gao & Schaffer, 1999 ▸). The maximum activity of maize alkaline α-galactosidase was detected in mature dehydrated, germinating and germinated seeds (Zhao *et al.*, 2006 ▸); ZmAGA1, with maximum catalysis at pH 7.5, was solely involved in seed germination. Elevated levels of alkaline α-galactosidase gene expression were detected when seed germination in maize was interrupted by heat, cold or dehydration (Zhao *et al.*, 2006 ▸) and when New Zealand spinach was grown under drought conditions (Hara *et al.*, 2008 ▸). A rice chloroplast alkaline α-galactosidase (OsAkαGal) has been suggested to exhibit a function in degrading digalactosyl diacylglycerol, a major glycolipid in the chloro­plast thylakoid membrane, during leaf senescence (Lee *et al.*, 2004 ▸, 2009 ▸).

The *Arabidopsis thaliana* (*Arabidopsis*) genome contains genes for three alkaline α-galactosidases (*At1g55740*, *At3g57520* and *At5g20250*), two RFO synthases (*At4g01970* and *At5g40390*) and four acidic α-galactosidases (*At3g26380*, *At5g08370*, *At5g08380* and *At3g56310*). Here, the proteins encoded by *At1g55740*, *At3g57520* and *At5g20250* are referred to as AtAkαGal1, AtAkαGal2 and AtAkαGal3, respectively (Lee *et al.*, 2004 ▸). A *BLAST* search showed that AtAkαGal3 is related to probable galactinol–sucrose galactosyltransferases from various species, such as *Arabidopsis lyrata* subsp. *lyrate* (XP_020876652), *Camelina sativa* (XP_010493135), *Capsella rubella* (XP_006287127), *Raphanus sativus* (XP_018444981), *Brassica oleracea* var. *oleracea* (XP_013612073.1) and *Prunus persica* (XP_020424207), with 76–94% sequence identity. Moreover, AtAkαGal3 shares sequence identities of less than 30% with all acidic α-galactosidases from *Arabidopsis* and other plants.

To date, only structures of acidic α-galactosidases have been reported, including rice acidic α-galactosidase (PDB entry 1uas; Fujimoto *et al.*, 2003 ▸), chicken α-*N*-acetylgalactos­aminidase (PDB entry 1ktb; Garman *et al.*, 2002 ▸) and human α-galactosidase (PDB entry 1r46; Garman & Garboczi, 2004 ▸). The structures of rice acidic α-galactosidase and the closely related chicken α-*N*-acetylgalactos­aminidase revealed a double-displacement catalytic mechanism and the mode of substrate binding (Fujimoto *et al.*, 2003 ▸; Garman *et al.*, 2002 ▸). However, no structure of any alkaline α-galactosidase or RFO synthase was available prior to this work. Here, we report structures of AtAkαGal3 and its D383A mutant in complex with various substrates and products, including the natural substrate/product stachyose, revealing four complete substrate-binding subsites (–1 to +3) at the catalytic site for catalysis. The dual-functional AtAkαGal3 was also discovered to exhibit stachyose synthase activity in this work; the new structure of AtAkαGal3 thus potentially serves as a model for the study of raffinose/stachyose synthases.

## 2. Methods

### 2.1. Cloning, expression and purification

Materials and chemicals were obtained from Sigma unless specified otherwise. *A. thaliana* L. Herynh plants (Col-0) were used in this study. *AtAk*α*Gal3* full-length open frame sequences (2273 bp) were obtained using database searches and sequence analyses using OsAkαGal as a query (Lee *et al.*, 2004 ▸). The S3 stage of senescent leaves containing about 30–50% chlorophyll compared with full-grown green rosette leaves (100% chlorophyll) was used for total RNA extraction. Chlorophyll determination was carried out as described previously (Lee & Collins, 2001 ▸). Total RNA was isolated using the method described previously (Chang *et al.*, 1993 ▸), and an Agilent 2100 Bioanalyzer (ThermoFisher, USA) was used to assess its quality and quantity. First-strand cDNA was synthesized from 1 µg of total RNA using the SuperScript III First-Strand Synthesis System, as described by the manufacturer (ThermoFisher, USA). A DNase (Promega, USA) digestion step was conducted prior to reverse transcription in a total volume of 20 µl. *AtAk*α*Gal3* full-length open frame sequences (2273 bp) containing KpnI and SalI sites were amplified from 1 µl cDNA solution by 30 cycles of denaturation at 94°C for 30 s, annealing at 55°C for 30 s and extension at 72°C for 1 min in 20 µl PCR reaction mixture. The primer pair used was At5gKpnIF2 (GATCTGGGTACCATGACGATTAAACCGGCGGT) and At5gnostopSalIR (CTGCAGGTCGACTAACTCAACTTGGATCAGA). The *AtAk*α*Gal3* full-length open frame sequences were cloned into the expression vector pET-30a(+) (Novagen) at KpnI and XhoI sites with a 6×His tag at the N-terminus and were transformed into *Escherichia coli* BL21(DE3) cells for expression. The bacteria were cultured at 37°C until the OD_600_ reached ∼0.6 prior to induction with isopropyl β-d-thiogalactopyranoside (IPTG; final concentration 0.15 m*M*) in Luria–Bertani (LB) medium at 20°C for 20 h with shaking, and were then harvested by centrifugation at 8000*g* for 25 min at 4°C. The cell pellet from 1 l culture was suspended in lysis buffer (35 ml) consisting of 50 m*M* Tris–HCl pH 8.0 and was subjected to cell disruption by ultrasonication using a pulse cycle of 2 s on and 3 s off with a total duration of 20 min at 40% energy on ice. The soluble protein extract was collected by centrifugation at 12 000*g* for 30 min at 4°C. The target AtAkαGal3 was purified using a nickel-immobilized metal ion-affinity chromatography (Ni-IMAC) column with a low imidazole concentration (50 m*M*), thus avoiding protein contamination at higher concentrations that affects crystallization, in 20 m*M* Tris–HCl buffer pH 7.8 containing 500 m*M* NaCl and was further dialyzed against 20 m*M* Tris–HCl buffer pH 8.2 containing 1 m*M* dithiothreitol (DTT) and 1 m*M* galactose overnight. The eluted AtAkαGal3 was concentrated and subjected to size-exclusion chromatography on an S200 column previously equilibrated with the abovementioned dialysis buffer. The target protein was then purified using the same buffer condition as the dialysis buffer to improve the purity.

Two AtAkαGal3 mutants, D383A and D447A, were generated by a site-directed mutagenesis method (QuikChange kit, Stratagene) using the pET-30a-AtAkαGal3 recombinant vector as a template. The forward primers for the mutants are as follows: D383A, 5′-GGACGGTGTGAAAGTGGCTGTGCAGTGTGTATTGG-3′; D447A, 5′-TGATTAGAGCATCAGATGCTTTCTATCCACGGGATCC-3′. The D383A and D447A mutants were confirmed by sequencing before expression. After Ni-IMAC purification, both mutants were dialyzed against the same dialysis buffer as wild-type AtAkαGal3 protein with and without galactose for various substrate and product complex studies. Selenomethionine-labeled AtAkαGal3 (SeMet-AtAkαGal3) protein was prepared following the method of Van Duyne (Doublié, 2007 ▸). Briefly, a single colony of recombinant AtAkαGal3 in *E. coli* BL21(DE3) cells was inoculated into 5 ml LB culture at 37°C for 12 h and then inoculated into 100 ml M9 medium in a 250 ml flask and cultured overnight. The bacterial cells from this overnight culture were collected and inoculated into 1 l M9 medium; a mixture of amino acids (100 mg lysine, phenylalanine and threonine; 50 mg isoleucine, leucine and valine; 60 mg SeMet) was added when the OD reached 0.6. After bacterial growth for a further 15 min, 0.15 m*M* IPTG was added to induce protein production. The rice raffinose synthase from *O. sativa* L. var. Nipponbare (XP_015621501) was overexpressed, purified and characterized using the protocol described previously (Li *et al.*, 2007 ▸).

### 2.2. Activity assay using thin-layer chromatography

The purified proteins, wild-type AtAkαGal3 and its D383A and D447A mutants, as well as rice raffinose synthase, at equal amounts of 4 × 10^−12^ mol were incubated with the substrate raffinose at a final concentration of 10 m*M* at 35°C for 1 h. Each protein reaction and standard oligosaccharides (galactose, sucrose, raffinose and stachyose) were spotted (2 µl) onto a silica thin-layer plate (Merck Millipore); the sample was developed with a solvent consisting of a mixture of chloroform, acetic acid and water in a 6:7:1 ratio by volume (Farag, 1978 ▸). The results of the reaction were examined by spraying ethanol containing sulfuric acid (10%) onto the plate and baking it on a hotplate until brown spots developed.

### 2.3. Crystallization and collection of X-ray data

The purified SeMet-AtAkαGal3, wild-type and mutant AtAkαGal3 protein fractions from size-exclusion chromatography were concentrated to 40–50 mg ml^−1^ in a buffer consisting of 20 m*M* Tris–HCl pH 8.2, 1 m*M* DTT with or without 1 m*M* galactose before crystallization, which was performed using a microbatch method. The protein crystals appeared in 2–3 months using the best crystallization condition (0.1 *M* ammonium sulfate, 0.3 *M* sodium formate, 0.1 *M* Tris pH 7.8, 10% PEG 2000) at 18°C. Among the crystallization trials with various AtAkαGal3 proteins, crystals of SeMet-AtAkαGal3, wild-type AtAkαGal3 and the D383A mutant (referred to in the following as D383A) in various substrate or product complexes could be obtained. No satisfactory crystals of the D447A mutant were obtained. In addition, without galactose neither wild-type nor mutant crystals could be obtained. Crystals of D383A–substrate complexes were obtained by co-crystallization and subsequent crystal soaking with various substrates, including galactinol, galactose, raffinose and stachyose (final concentration 5 m*M*), in the reservoir solution at 25°C for 10 min.

The crystals were harvested, transferred to reservoir solution containing glycerol (20%) as a cryoprotectant and immediately cooled with liquid nitrogen for data collection. The X-ray diffraction experiments were performed at various wavelengths ranging from 0.9 to 1.0 Å on synchrotron beamlines TPS 05A (with a Rayonix MX300HS detector) and TLS 15A (with a Rayonix MX300HE detector) at the National Synchrotron Radiation Research Center (NSRRC) in Taiwan and on BL44XU (with a Dectris EIGER X 16M detector) at SPring-8 in Japan. The data sets for SeMet-AtAkαGal3, AtAkαGal3–galactose, D383A–galactose, D383A–stachyose and D383A–galactose–sucrose collected on the TPS 05A and TLS 15A beamlines were processed using *HKL*-2000 (Otwinowski & Minor, 1997 ▸) and the D383A–galactinol and D383A–rafffinose data sets collected on the BL44XU beamline were processed with *XDS* (Kabsch, 2010 ▸).

### 2.4. Structure determination and refinement

The initial crystal structure of wild-type AtAkαGal3 was determined by the selenomethionine multi-wavelength anomalous diffraction (Se-MAD) phasing method. 29 selenomethionine sites were first located using *SOLVE* (Terwilliger & Berendzen, 1999 ▸) to generate reasonable initial phases with a figure of merit of 0.34 for data in the resolution range 30–3.0 Å. The phases were further calculated and improved with *AutoSol* (Zhao *et al.*, 2006 ▸) in *Phenix* and the direct phase selection method (Chen *et al.*, 2014 ▸). A model of most of the structure was autobuilt with *ARP*/*wARP* (Langer *et al.*, 2008 ▸), and further model building and adjustment were undertaken with *Coot* (Emsley *et al.*, 2010 ▸). The structures of the D383A mutant in its various complexes were determined by molecular replacement with *MOLREP* (Vagin & Teplyakov, 2010 ▸) from the *CCP*4 suite (Winn *et al.*, 2011 ▸) using the previously solved structure of wild-type AtAkαGal3 as a model. All refinements were performed with *REFMAC* (Murshudov *et al.*, 2011 ▸) from the *CCP*4 suite. The structural models of galactose, galactinol, raffinose and starchyose were obtained from *eLBOW* (Moriarty *et al.*, 2009 ▸) in *Phenix* before building and refinement.

#### 2.4.1. Model validation

The final refined structures of wild-type AtAkαGal3 and the various D383A–substrate and D383A–product complexes were validated with *MolProbity* (Williams *et al.*, 2018 ▸). The ligand-binding interaction at the catalytic binding site of the AtAkαGal3 structures was analyzed by *PLIP* (Salentin *et al.*, 2015 ▸).

#### 2.4.2. Accession codes

All of the coordinates and structural factors in this work have been deposited in the Protein Data Bank as PDB entries 7exf, 7exg, 7exh, 7exj, 7exr and 7exq for WT AtAkαGal3–galactose, D383A–galactose, D383A–galactinol, D383A–rafffinose, D383A–stachyose and D383A–galactose–sucrose, respectively.

## 3. Results and discussion

### 3.1. Protein purification

Both the recombinant wild-type AtAkαGal3 and the D383A mutant were passed through an Ni-IMAC column and a size-exclusion column (S200) for purification. All purified AtAkαGal3 and mutant proteins showed a single band corresponding to a molecular mass of ∼82 kDa on SDS–PAGE. The target proteins eluted from size-exclusion chromatography at a molecular mass in the range 80–100 kDa, indicating that the biological unit of AtAkαGal3 is a monomer.

### 3.2. Hydrolytic activity assay

A hydrolytic reaction mixture containing AtAkαGal3 and its natural substrate raffinose was incubated and subsequently analyzed by thin-layer chromatography (TLC; Supplementary Fig. S1). For a comparison of activity, a similar assay was simultaneously performed with purified recombinant rice raffinose synthase (*O. sativa* L. var. Nipponbare; Li *et al.*, 2007 ▸). After the hydrolysis reactions with rice raffinose synthase and with AtAkαGal3, the TLC plate showed that the raffinose substrate was hydrolyzed to galactose and sucrose (lane 5) and to galactose, sucrose and stachyose (lane 6), respectively, while excess raffinose substrate remained. The results indicate that the hydrolase activity of AtAkαGal3 is greater than that of rice raffinose synthase towards raffinose as a substrate. Besides hydrolase activity, AtAkαGal3 exhibits stachyose synthase activity by transferring a galactose moiety to the raffinose substrate, in which both the galactose donor and acceptor are raffinose. Moreover, the TLC results showed that AtAkαGal3 appears to be incapable of synthesizing raffinose as a raffinose synthase according to an activity comparison between AtAkαGal3 and rice raffinose synthase with galactinol as the galactose donor and sucrose as the acceptor (data not shown). The activity assay thus shows that alkaline α-galactosidase exhibits a dual function of hydrolase and stachyose synthase activities.

### 3.3. Overall structure of *Arabidopsis* alkaline α-galactosidase

The crystal structure of wild-type AtAkαGal3 was first solved *ab initio* by the selenomethionine multi-wavelength anomalous diffraction (Se-MAD) method at a resolution of 2.8 Å and was subsequently refined to a high resolution of 2.17 Å using the native crystal with the best diffraction quality, which contains two molecules in the asymmetric unit (Supplementary Fig. S2*a* ). The crystal structures of complexes of the D383A mutant with galactose, galactinol, raffinose and stachyose were subsequently determined using the wild-type AtAkαGal3 structure as a search model by the molecular-replacement method (Table 1 ▸). The crystal structure of AtAkαGal3 comprises 724 of a total of 749 amino acids that fold into three domains: N-terminal, catalytic and C-terminal (Supplementary Fig. S2*b* ). The first four residues are absent due to a lack of electron density. The N-terminal domain (residues 5–187) consists of 13 antiparallel β-strands connected by variable loops with an α-helix at the end. The catalytic domain (residues 206–518) folds into an (α/β)_8_-barrel structure with eight parallel β-strands placed around a central axis and surrounded by eight α-helices, which can be seen in α-galactosidases of both the GH27 and GH36 families. The C-terminal domain comprises 16 β-strands that form two Greek-key motifs comprising residues 537–653 and 654–749, respectively, which are presumably involved in structural stability. Two regions of residues, 103–119 at the N-terminal domain and 254–263 at the catalytic domain, are disordered without electron density, possibly due to loop flexibility on the protein surface (Supplementary Fig. S2*b* ).

### 3.4. Protein sequence alignment

A *BLAST* search with the amino-acid sequence of AtAkαGal3 shows that the highest sequence similarities of 75–94% correspond to various probable galactinol–sucrose galactosyltransferases from different species, such as *A. lyrata* subsp. *lyrate* (94%), *C. rubella* (92%), *C. sativa* (91%), *R. sativus* (87%), *O. sativa* L. var. Nipponbare, *B. oleracea* (87%) and *P. persica* (76%). However, Lee *et al.* (2009 ▸) analyzed 31 plant α-galactosidases and RFO synthases based on sequence alignments and identities and found that they could be classified into acidic α-galactosidases, alkaline α-galactosidases and RFO synthases, among which AtAkαGal3 belongs to the alkaline α-galactosidase group. Some structures of acidic α-galactosidases have been reported, including α-galactosidase from *Thermotoga maritima* (Ren *et al.*, 2018 ▸), chicken α-*N*-acetylgalactos­aminidase (Garman *et al.*, 2002 ▸), rice α-galactosidase (Fujimoto *et al.*, 2003 ▸), human α-galactosidase (Garman & Garboczi, 2004 ▸) and *Trichoderma reesei* α-galactosidase (Golubev *et al.*, 2004 ▸), which share low sequence identities of 32%, 29%, 27%, 25% and 24%, respectively, with AtAkαGal3, whereas other α-galactosidases from *Nicotiana benthamiana* (Kytidou *et al.*, 2018 ▸), *Bacteroides fragilis* (Joint Center for Structure Genomics, unpublished work) and *Saccharomyces cerevisiae* (Fernández-Leiro *et al.*, 2010 ▸) show no reasonable identity to AtAkαGal3.

### 3.5. The catalytic residues and binding sites for various natural substrates

A sequence alignment of acidic α-galactosidases from rice (Fujimoto *et al.*, 2003 ▸), chicken (Garman *et al.*, 2002 ▸), human (Garman & Garboczi, 2004 ▸), *N. benthamiana* (Kytidou *et al.*, 2018 ▸), *B. fragilis*, a Gram-negative bacterium (Joint Center for Structure Genomics, unpublished work), *S. cerevisiae* (Fernández-Leiro *et al.*, 2010 ▸) and *T. reesei* (Golubev *et al.*, 2004 ▸) with known structures indicates the presence of two conserved motifs K*X*D and R*xxx*D, where *X* can be Y, F, L or I and *x* is any variety of amino acid. The aspartic acid residues in the K*X*D and R*xxx*D motifs serve as the nucleophile and the catalytic acid (Fujimoto *et al.*, 2003 ▸; Garman *et al.*, 2002 ▸), respectively (Supplementary Fig. S3). A sequence comparison of AtAkαGal3 with acidic α-galactosidases further confirmed that Asp383 and Asp447 are presumably critical residues that serve as the nucleophile and the catalytic acid/base, respectively. In addition, a sequence alignment of AtAkαGal3 with alkaline α-galactosidases from tomato (AF512549) and melon (AY114164), seed imbibition protein from *Arabidopsis* (AtAkαGal1) and RFO synthases from japonica rice (XP_015621501), cucumber (AAD02832), soybean (XP_006576826), yoshino cherry (PQQ02596) and clementine (XP_006444535) shows these two residues, Asp383 and Asp447 in AtAkαGal3, to be conserved in these motifs: _381_KVD_383_, except for raffinose synthase from cucumber where the valine is replaced by isoleucine, and _443_R*xx*DD_447_ in the catalytic domain. Based on the alignment, Arg443 and Ala444 of the _443_RASDD_447_ motif in AtAkαGal3 and most alkaline α-galactosidases are evidently conserved compared with those in RFO synthases (the R*xxx*D motifs contain the nucleophile and catalytic acid; Fujimoto *et al.*, 2003 ▸; Garman *et al.*, 2002 ▸; Supplementary Fig. S4).

To confirm that these two residues, Asp383 and Asp447 in AtAkαGal3, play important roles in catalysis, we generated two single mutants D383A and D447A by replacing Asp383 and Asp447 with alanine by site-directed mutagenesis. The TLC results showed that the hydrolase activity of both mutants towards raffinose was abolished (data not shown).

### 3.6. Interactions of galactose at the binding site of wild-type AtAkαGal3 and the D383A mutant

**Figure 1:**
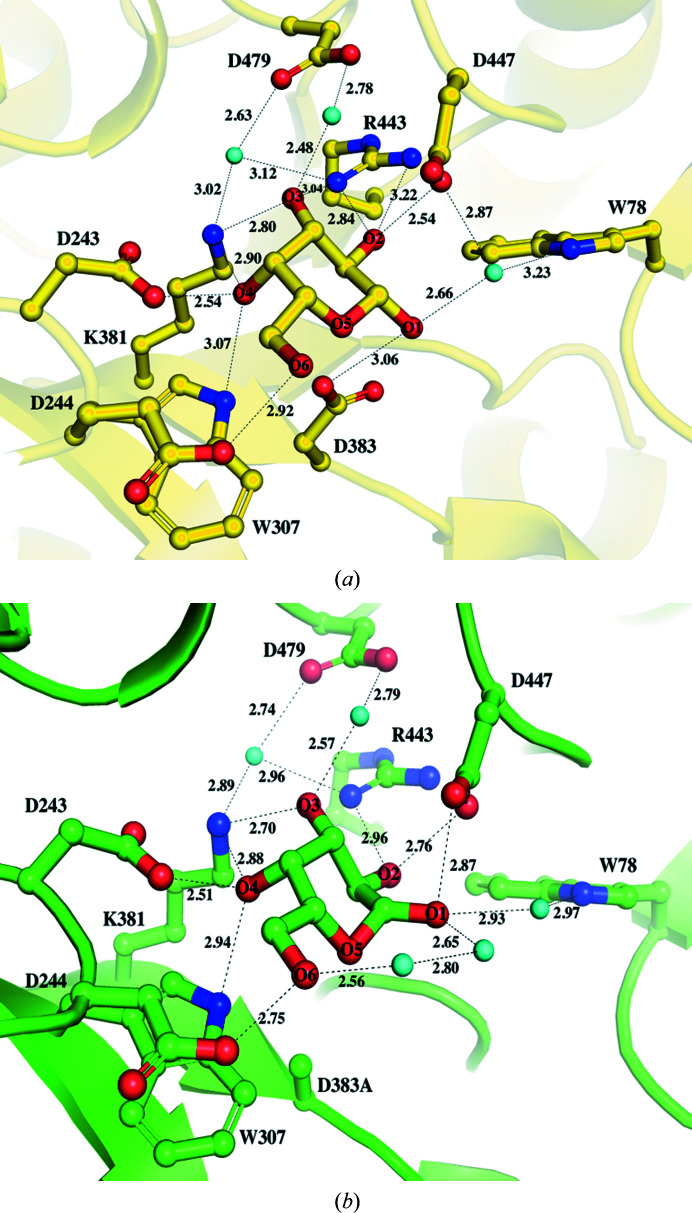
Active site of the crystal structure of AtAkαGal3. (*a*) The crystal structure of wild-type AtAkαGal3. The detailed interactions of galactose (yellow sticks) in a β-anomeric form with residues (yellow sticks) at the −1 subsite are shown. OH1 of galactose makes a hydrogen-bond interaction with the side chain of Asp383 and a water molecule (cyan sphere), with distances of 3.06 and 2.66 Å, respectively. Three water molecules (cyan spheres) help to stabilize the galactose moiety through water-mediated hydrogen-bonding interactions with Trp78, Lys381, Arg443, Asp447 and Asp479. (*b*) A galactose bound at the catalytic site of the D383A mutant forms an interaction network with residues similar to that in wild-type AtAkαGal3, except that there is a direct interaction between OD2 of Asp447 and O1 of the galactose moiety; two additional water molecules (cyan spheres) help to stabilize O1 and O6 of galactose as Asp383 is replaced by alanine.

Before the crystallization of wild-type AtAkαGal3, galactose was added to the protein solution via dialysis in order to obtain suitable crystals. Our first solved structure of wild-type AtAkαGal3 was thus in complex with galactose at a resolution of 2.17 Å. The galactose moiety, with a chair conformation, is located in the −1 subsite of the catalytic binding site (Fig. 1 ▸ *a*). The structure of the AtAkαGal3–galactose complex shows that the OH group at C1, the anomeric C atom, of the galactose moiety is in a β-anomeric form and points towards the bottom side of the catalytic binding site, forming a direct hydrogen bond (distance 3.06 Å) to the nucleophile Asp383 and to the OD1 group of the acid/base catalytic residue Asp447 through a water molecule, with distances of 2.66 and 2.87 Å, respectively. Moreover, the OD1 group of Asp447 makes a strong hydrogen bond (distance 2.54 Å) to the OH2 group of the galactose. The other hydroxyl groups of the galactose are also stabilized by numerous hydrogen-bond interactions with various surrounding side chains of the residues at the −1 binding site, such as Arg443, Lys381, Asp243, Asp244 and Trp307. Furthermore, three water molecules help to stabilize the galactose moiety through water-mediated hydrogen-bonding interactions with Trp78, Lys381, Arg443, Asp447 and Asp479.

The structure of the D383A mutant in complex with galactose was also determined at a resolution of 2.05 Å (Fig. 1 ▸ *b*). Interestingly, superposition of the two galactose moieties at the catalytic binding sites of the wild-type AtAkαGal3–galactose and D383A–galactose complex structures shows that the galactose moiety in the D383A mutant exhibits a chair form with a slight distortion; the O1 hydroxyl group at the C1 carbon is in an α-anomeric form (Supplementary Fig. S5). This particular structural form thus places the O1 hydroxyl group of the galactose in a position nearer the OD2 group of Asp447 to form a direct hydrogen-bond interaction with a distance of 2.87 Å. This effect might be due to the loss of an interaction with the carboxyl group of Asp383, which is replaced by alanine with a short side chain. Except for O1 at the anomeric C atom, which exhibits a conformational alteration from a β-form to an α-form, the positions of the remaining C atoms and hydroxyl groups of galactose are conserved and make unchanged interactions around the catalytic site compared with the wild-type enzyme. In the D383A structure, two additional water molecules interacting with O1 and O6 of galactose help to stabilize the galactose moiety compared with the wild-type AtAkαGal3 structure. Using the Cremer–Pople calculator, the galactose molecules complexed in the D383A and D383D mutants show Cremer–Pople parameters φ, θ, *Q* of 88.829°, 24.021°, 0.713 and 123.014°, 6.867°, 0.646, respectively, which might indicate an induced fit of the substrate to push it towards the transition state.

The residues involved in product-binding interactions with the galactose moiety at the −1 subsite of AtAkαGal3 are similar to those in rice acidic α-galactosidase (Fujimoto *et al.*, 2003 ▸), except for Trp78 in AtAkαGal3, which is structurally related to Trp164 of rice acidic α-galactosidase and to Trp65 of *T. maritima* α-galactosidase (Ren *et al.*, 2018 ▸), which positions its side chain farther away from the galactose moiety. Trp164 of rice acidic α-galactosidase, which is located on the loop at the end of the β5 strand, directly interacts with the O1 hydroxyl group of the galactose at the −1 subsite and is a conserved residue among acidic α-galactosidases from plants, bacteria and yeast, suggesting that it plays a role in recognizing the galactose substrate moiety (Fujimoto *et al.*, 2003 ▸). In contrast, Trp78 of AtAkαGal3, which is located in a short β-turn (residues 75–78) between two β-strands of the N-terminal domain, contributes part of the catalytic binding site together with the catalytic domain. Except for water-mediated interactions with the O1 hydroxyl group of the galactose and the side chain of Asp447, Trp78 seems to make no direct interactions with the galactose moiety at the −1 subsite in AtAkαGal3, but rather to make a major interaction at the +1 subsite, which is discussed in the following section.

### 3.7. Interactions of galactinol in the binding site of the D383A mutant

**Figure 2:**
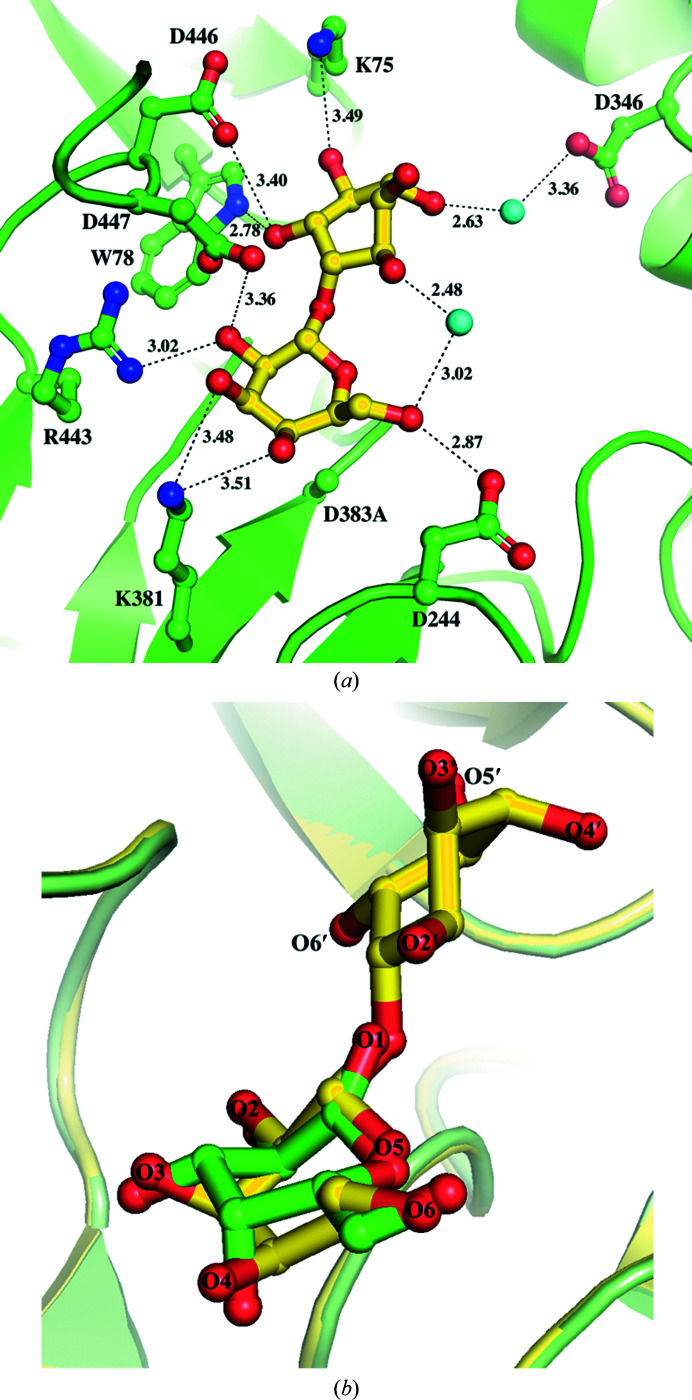
Interactions of galactinol at the active site of the D383A mutant compared with galactose binding to Asp383 in the wild type. The structure of D383A in complex with galactinol is shown. (*a*) The galactosyl moiety of galactinol (yellow sticks) is bound at the −1 subsite. OD2 of Asp447 makes a direct interaction with O2 of the galactosyl moiety at a distance of 3.36 Å. Lys75, Trp78, Asp346, Asp446 and Tyr449 (green sticks) are involved in stabilization of the myo-inositol moiety by hydrogen-bonding interactions at the +1 subsite. Water molecules are shown as cyan spheres. (*b*) The carbon ring of the galactosyl moiety (yellow) of galactinol is distorted from that of galactose (green) bound to Asp383; the O3 and O4 groups hence interact with a different residue, Lys381, at the catalytic site as shown in (*a*).

The structure of D383A in complex with galactinol was determined at a resolution of 2.63 Å from D383A crystals by a combination of co-crystallization and soaking with galactinol as described in Section 2. The 5*R*-OH group (O6′) of the myo-inositol moiety of galactinol at the +1 subsite is stabilized by the NE1 group of Trp78 together with OD1 of Asp446 by hydrogen bonds. In addition, 4*S*-OH is stabilized by the NZ group of Lys75 by hydrogen bonds (Fig. 2 ▸ *a*). The conformation of the galactosyl moiety of galactinol differs from that of galactose bound to wild-type AtAkαGal3 and the D383A mutant (Fig. 2 ▸ *b*), but the galactosyl moiety remains stabilized at the −1 subsite by mostly the same residues, including Asp447, Arg443, Lys381 and Asp244, but not Asp243 and Trp307, making interactions with galactose. Two water molecules are located in the catalytic binding site, one of which interacts with the galactosyl moiety and the other with the myo-inositol moiety and the side chain of Asp346, to help stabilize the galactinol. This Asp346 residue seems to play a major role in galactinol binding at the +1 subsite (Fig. 2 ▸ *a*).

### 3.8. Interactions of raffinose in the binding site of the D383A mutant

**Figure 3:**
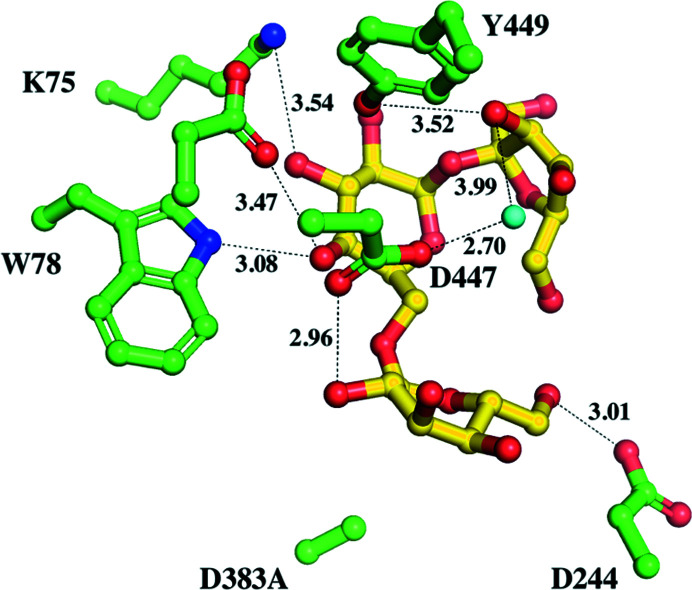
The active-site structure of the complex of D383A with raffinose (yellow sticks). Various residues (green sticks) are involved in binding inter­actions with glucose at the +1 subsite and fructose at the +2 subsite, as well as the galactosyl moiety at the −1 subsite. The catalytic residue Asp447 and the D383A mutation are also shown. One water molecule (cyan sphere) is located at the catalytic binding site to help stabilize the fructose moiety through hydrogen bonding.

The crystal structure of D383A in complex with raffinose was determined at a resolution of 2.47 Å. Lys75, Tyr78, Asp446 and Tyr449 help to stabilize the glucose moiety of the raffinose substrate at the +1 subsite (Fig. 3 ▸). The NE1 group of Trp78 and OD1 of Asp446 form hydrogen bonds to O4 of the hydroxyl group with distances of 3.08 and 3.47 Å, respectively. NZ of the Lys75 side chain stabilizes O3 of the hydroxyl group through a hydrogen bond with a distance of 3.54 Å. Tyr449 stabilizes the glucose at the +1 subsite and the fructose moiety at the +2 subsite of raffinose via hydrogen bonds from its side-chain OH group to the O3 hydroxyl groups of both galactose and fructose with distances of 3.52 Å (Fig. 3 ▸). One water molecule that forms a hydrogen bond (distance of 2.70 Å) to the side chain of Asp447 seems to be involved in stabilization of the fructose moiety through weak hydrogen bonding. The interactions of the residues at the −1 subsite with the galactosyl moiety of raffinose are similar to those with the galactosyl moiety of galactinol bound in the D383A complex.

### 3.9. Interactions of the galactose and sucrose products of hydrolyzed raffinose in the binding site

**Figure 4:**
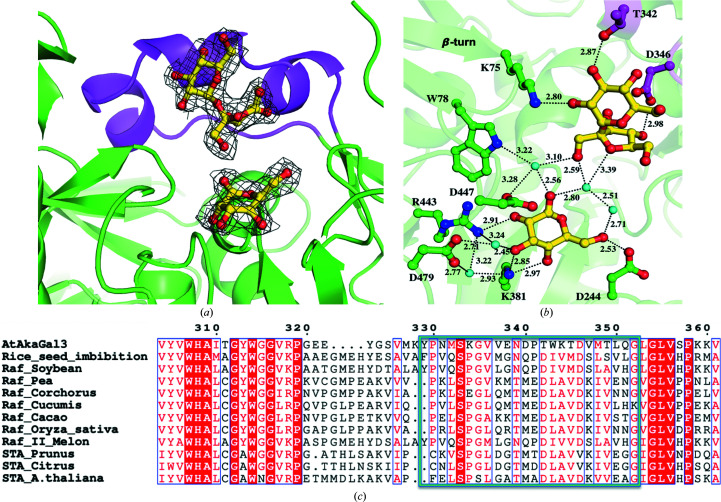
The electron density of galactose and sucrose products at the active site and sequence alignment of the loop region. (*a*) Both the galactose and sucrose products (yellow sticks) are clearly revealed with electron densities (gray mesh) at the −1 subsite and the secondary product-binding site, respectively, after co-crystallization of D383A and raffinose for two months. The secondary product-binding site is near the surface of the catalytic binding cavity, which is formed in part by a unique loop with three short helices (magenta) and a β-turn (residues 75–78). (*b*) The interaction network with distances between galactose, sucrose (yellow sticks) and residues (green and magenta sticks) is shown. Water molecules are presented as cyan spheres. (*c*) A sequence alignment shows that the loop region (residues 329–352 in the green box) exists exclusively in AtAkαGal3 and possibly in the RFO synthase family from different plants, including rice (ABF99470; Rice_seed_imbibition), soybean (XP_006576826; Raf_Soybean), pea (RFS_PEA; Raf_Pea), jute (OMO49898; Raf_Corchorus), cucumber (AAD02832; Raf_Cucumis), cacao (EOY02480; Raf_Cacao), japonica rice (XP_015621501; Raf_Oryza sativa), melon (NP_001284472; Raf_II_Melon), yoshino cherry (PQQ02596; STA_Prunus), clementine (XP_006444535; STA_Citrus) and *Arabidopsis* (NP_192106; STA_*A. thaliana*). Residue numbers are based on AtAkαGal3.

During the crystallization of the D383A–raffinose complex, co-crystallization of the D383A mutant and the raffinose substrate was occasionally set up for over two months. Unexpectedly, we observed both the galactose and sucrose products in the catalytic binding site and an additional cavity in the crystal structure after a sufficient reaction time (Fig. 4 ▸ *a*). The D383A mutant seems to be capable of gradually hydrolyzing the raffinose substrate into galactose and sucrose products that remain in the catalytic site. The D383A mutant in complex with hydrolyzed raffinose reveals a secondary product-binding site for sucrose; this site is stabilized and formed in part by a unique short loop (residues 329–352) composed of three short helices at the surface of the catalytic pocket, which equip Thr342 and Asp346 to interact with the glucose and fructose moieties of sucrose through hydrogen bonds (Fig. 4 ▸ *b*). This distinct extra loop (residues 329–352) in AtAkαGal3 can also be found in raffinose/stachyose synthases based on sequence alignment (Fig. 4 ▸ *c*), but not in acid α-galactosidase-related proteins. Lys75 from a β-turn (residues 75–78) in the N-terminal domain also helps to stabilize the glucose and fructose moieties of sucrose by hydrogen bonds with a distance of 2.80 Å. In addition, Trp78 and Asp447 help to stabilize both the sucrose product and the galactose moiety at the −1 subsite through a water molecule (Fig. 4 ▸ *b*).

### 3.10. Interactions of stachyose in the binding site of the D383A mutant

**Figure 5:**
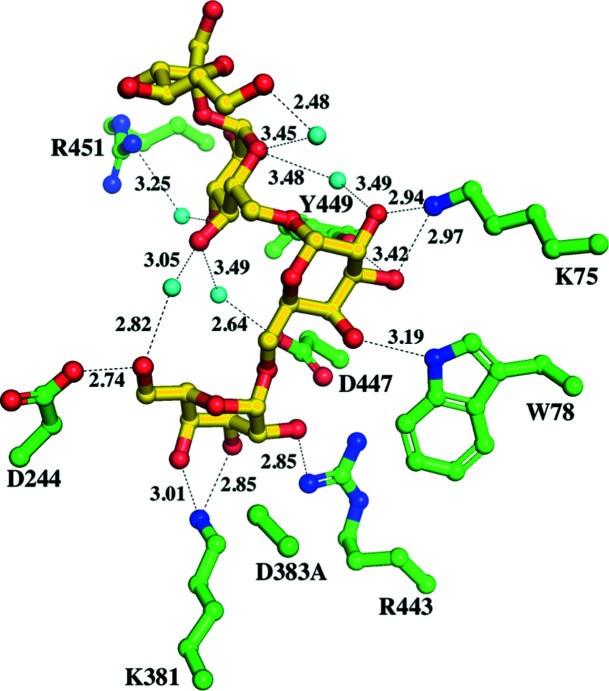
The active-site structure of D383A in complex with stachyose. The complexed structure reveals the complete subsites (–1, +1, +2 and +3) in the catalytic binding site of AtAkαGal3. The glucose in the +2 subsite, stabilized by Arg451, Tyr215 and Trp211 through water-mediated hydrogen-bond interactions, is located nearly at the edge of the pocket cavity. The binding interaction of fructose in the +3 subsite, which is located at the outer edge of the cavity, is utilized only by Asp346 (magenta sticks) and one water molecule at the outer edge of the cavity. Stachyose, protein residues and water molecules are shown as yellow sticks, green sticks and cyan spheres, respectively.

The crystal structure of the complex of D383A with stachyose determined at a resolution of 2.0 Å further reveals the complete subsites (–1, +1, +2 and +3) of AtAkαGal3 (Fig. 5 ▸). The galactose at the +1 subsite of stachyose is stabilized by hydrogen bonds to both NE of the Lys75 side chain and NH1 of the Trp78 side chain with distances of ∼3 Å. Even though Trp77 makes no direct interaction with galactose at the +1 subsite, it helps to stabilize galactose through a water molecule. The extensive water network through the side chain of Arg451 helps to stabilize the glucose moiety at the +2 subsite through a water molecule. In addition, water-mediated hydrogen-bond interactions greatly stabilize both the glucose and fructose moieties at the +2 and +3 subsites.

### 3.11. Structural and sequence comparison explain the dual function of AtAkαGal3

The GH36 family contains enzymes, including RFO synthases and alkaline α-galactosidases, that generally exhibit transglycosylation and hydrolase activities. The first reported α-galactosidase structure was that of *T. maritima* α-galactosidase (Ren *et al.*, 2018 ▸). Structural alignment of *T. maritima* α-galactosidase with the related GH27 family enzymes shows that *T. maritima* α-galactosidase and rice acidic α-galactosidase (Fujimoto *et al.*, 2003 ▸) share the greatest structural similarity. A structural comparison of the (α/β)_8_-barrel domain of our alkaline α-galactosidase AtAkαGal3 (residues 201–531) with those of rice acidic α-galactosidase (residues 6–271) and *T. maritima* α-galactosidase (residues 165–481) shows poor structural similarity, with r.m.s.d.s of 3.74 and 11.37 Å, respectively. The structure of wild-type AtAkαGal3 shows that the catalytic residues Asp383 and Asp447 are separated by approximately 6.7 Å, which is also generally found for the double-replacement retaining mechanism in members of clan D of the GH27 enzymes, with an average distance of 6.5 Å, and in *T. maritima* α-galactosidase, with a distance of 6.3 Å (Comfort *et al.*, 2007 ▸). A structural alignment of the (α/β)_8_-barrel domains of AtAkαGal3, rice acidic α-galactosidase and *T. maritima* α-galactosidase based on the AtAkαGal3 structure shows that most catalytic binding residues at the −1 subsite are conserved. In addition, Trp78 of AtAkαGal3 is not part of the (α/β)_8_-barrel of the catalytic domain like the corresponding Trp of rice acidic α-galactosid­ase, but is located in the N-terminal domain like Trp65 of *T. maritima* α-galactosidase. Moreover, Lys75 and Trp78 in the N-terminal domain share hydrogen-bond interactions that stabilize the second galactose moiety in the +1 subsite (Table 2 ▸).

**Figure 6:**
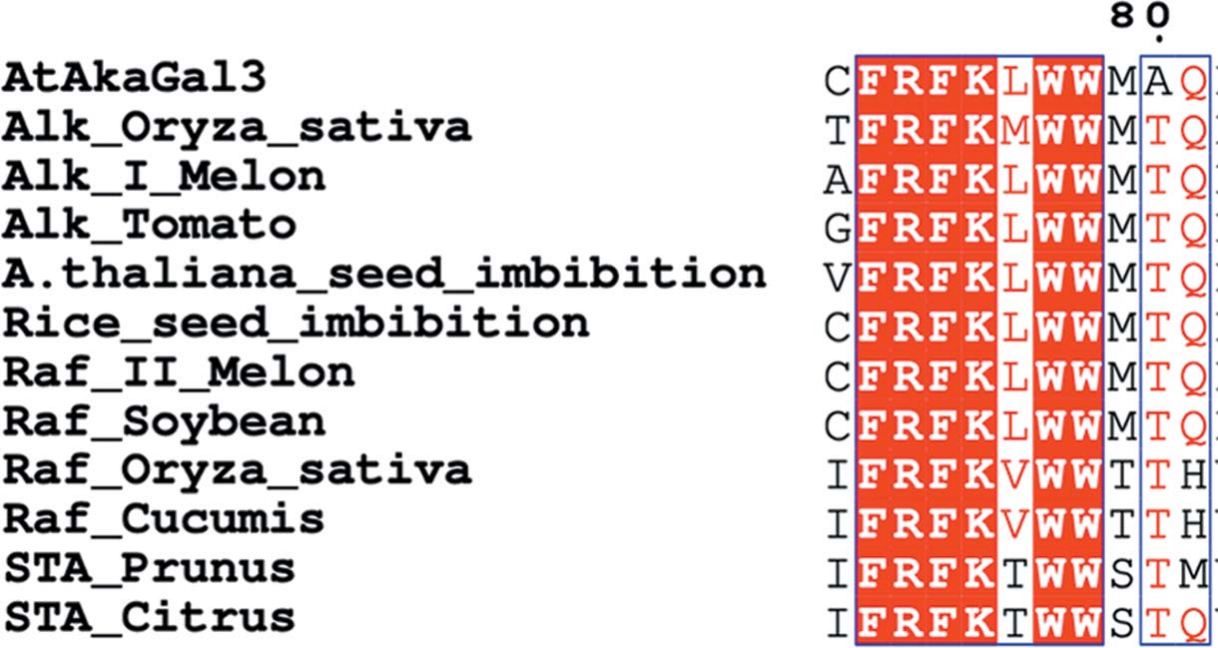
Sequence alignment of the WW box region of AtAkαGal3 with related enzymes from plant sources. The sequence alignment of alkaline α-galactosidases from rice (AF251068; Alk_Oryza_sativa), melon (AAM75139; Alk_I_Melon) and tomato (NP_001234763; Alk_Tamato), seed imbibition proteins from *Arabidopsis thaliana* (AtAkαGal1; A.thaliana_seed_imbibition) and rice (ABF99470; Rice_seed_imbibition) and raffinose/stachyose synthases from melon (NP_001284472; Raf_II_Melon), soybean (XP_006576826; Raf_Soybean), rice (XP_015621501; Raf_Oryza_sativa), cucumber (AAD02832; Raf_cucumis), yoshino cherry (PQQ02596; STA_Prunus) and clementine (XP_006444535; STA_Citrus) shows a conserved WW box region (FRSK_75_ *x* **W**_77_ **W**_78_) among alkaline α-galactosidases and the RFO synthases. The sequence numbering refers to AtAkαGal3.

A sequence alignment (*ESPript*3.0; Robert & Gouet, 2014 ▸) of plant alkaline α-galactosidases with plant raffinose and stachyose synthases shows that the FRSK_75_ *x*W_77_W_78_ region (referring to AtAkαGal3) located at the β-turn between two β-strands of the N-terminal domain is conserved among these families (Fig. 6 ▸). Furthermore, two residues, Arg451 and Tyr449 of AtAkαGal3, which are also found in raffinose and stachyose synthases, help to stabilize the binding of the galactose moiety at the +2 subsite through water molecules and directed interaction, respectively. These observations from both structural and sequence alignments lead to the possibility of a dual function of the alkaline α-galactosidase AtAkαGal3, as the identified residues involved in product and substrate binding at the −1, +1 and +2 subsites of the alkaline α-galactosidase AtAkαGal3 are identical to the functional residues in both the GH27 and GH36 families as well as the raffinose and stachyose synthase families. However, the structure of D383A complexed with stachyose shows that water molecules seem to be important to help to stabilize the fructose moiety at the +3 subsite (Table 2 ▸).

### 3.12. N-terminal domain and catalytic site of AtAkαGal3

Our work confirms that the N-terminal domain of the alkaline α-galactosidase AtAkαGal3 is important for protein function because it contributes part of the core (α/β)_8_-barrel catalytic site for substrate binding. The C-terminal domain helps to stabilize this structural architecture through inter­actions with both the N-terminal domain and the (α/β)_8_-barrel domain. Sequence alignment indicates that the RFO synthases might also use their N-terminal domain for a similar function. The stachyose bound in the catalytic pocket of AtAkαGal3 shows that the depth and dimensions of the binding pocket can accommodate four sugar moieties, whereas the catalytic binding sites of the acidic α-galactosidases rice α-galactosidase and *T. maritima* α-galactosidase are relatively shallow and are likely to accommodate only one galactose. Moreover, the extra loop (residues 329–352) that only exists in the alkaline α-galactosidase AtAkαGal3 provides two key residues Asp346 and Thr342 to help stabilize the fructose and glucose moieties of the sucrose product in the +2 and +3 subsites, respectively (Figs. 4 ▸ and 7 ▸).

**Figure 8:**
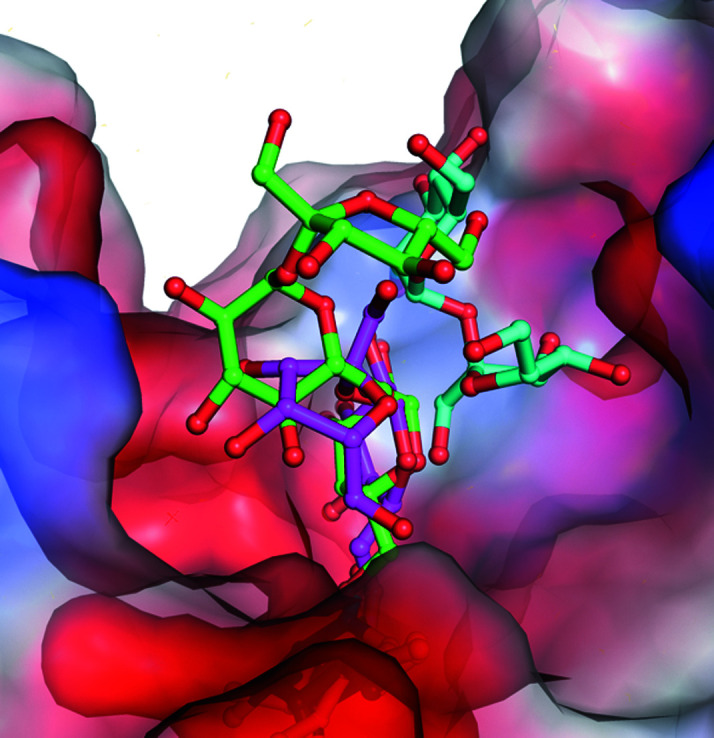
Active-site structures of D383A in complex with raffinose. The structures of D383A in complexes with raffinose (magenta), with stachyose (green) and with hydrolyzed raffinose, *i.e.* galactose and sucrose (cyan), in the catalytic binding pocket shown with the electrostatic surface indicate that the wider binding-site pocket of the alkaline α-galactosidase AtAkαGal3 provides a new secondary product-binding site to stabilize the sucrose moiety (cyan).

Our D383A mutant can gradually hydrolyze raffinose into galactose and sucrose. A structural superimposition of D383A in complex with raffinose, with stachyose and with hydrolyzed raffinose in the catalytic binding pocket shows that the wider binding-site pocket of AtAkαGal3 provides a secondary product-binding site to stabilize the sucrose after raffinose has been hydrolyzed into galactose and glucose. This product-binding site contains Thr342 and Asp346 on a short loop (residues 329–352) that interact with the glucose and fructose moieties of sucrose through hydrogen bonds. Lys75 in the N-terminal domain also helps to stabilize the glucose moiety of sucrose by hydrogen bonding (Fig. 8 ▸).

### 3.13. Dual function of AtAkαGal3

We compared the hydrolase activities of the alkaline α-galactosidase AtAkαGal3 and rice (*O. sativa* L. var. Nipponbare) raffinose synthase towards only the natural substrate raffinose, without galactinol, which serves as a galactose donor for raffinose or stachyose synthase activity. The TLC assay showed that AtAkαGal3 exhibits a greater hydrolase activity towards the raffinose substrate considering the TLC spot intensities of the galactose and sucrose products. AtAkαGal3 can also produce a stachyose product without galactinol as a galactose donor. This observation indicates that AtAkαGal3 seems to act with the transferase mechanism observed for fructosyl transferases and xyloglucan endotransferases. Our crystal structures of wild-type AtAkαGal3 and its D383A mutant in complex with galactose and with hydrolyzed raffinose, *i.e.* galactose and sucrose, reveal one water molecule at the conserved position near the anomeric C atom and Asp447. This water is replaced by O4 of galactose in the +1 subsite of raffinose and stachyose in the complexes of D383A with raffinose and stachyose. The only difference between the hydrolase and transferase reactions is the acceptor type after the removal of the terminal glycosyl moiety. The acceptor for a hydrolase is a water molecule, whereas a transferase uses a saccharide or an oligosaccharide, such as sucrose, raffinose or stachyose, as the acceptor (Henrissat *et al.*, 2001 ▸; Carmi *et al.*, 2003 ▸; Chuankhayan *et al.*, 2010 ▸). In AtAkαGal3, the conserved water molecule identified above might thus serve as an acceptor in the hydrolase step, releasing galactose as a product, while sucrose and raffinose act as an acceptor for the transferase mechanism, releasing raffinose and stachyose as products. In addition, the unique extra loop (residues 329–352) in AtAkαGal3, which contributes to the depth enhancement of the catalytic pocket, presumably supports the transferase mechanism to help to stabilize the oligomer products.

### 3.14. Catalytic and synthetic mechanism

**Figure 9:**
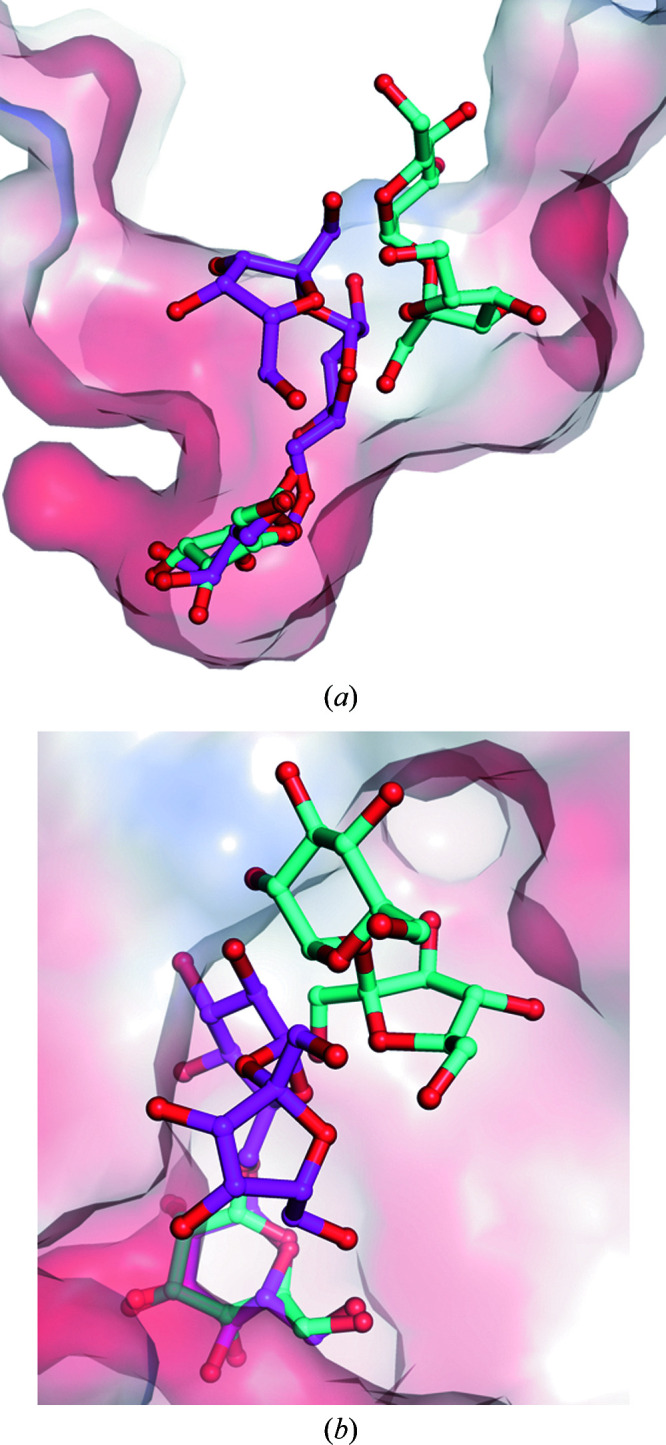
The active site for galactose and the secondary active site for sucrose. (*a*) A side view of the superimposed substrate raffinose (magenta) and products galactose and sucrose (cyan) from hydrolyzed raffinose in the catalytic binding pocket shown as an electrostatic surface. An empty space is found when the substrate raffinose is bound; this space is then occupied by the sucrose product after raffinose has been hydrolyzed. (*b*) A top view clearly shows that after raffinose has been hydrolyzed the sucrose product is flipped 180° and occupies the secondary product-binding site.

**Figure 10:**
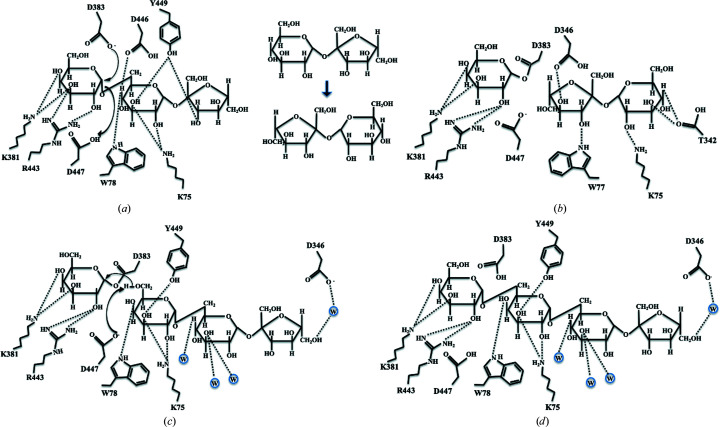
The proposed catalytic mechanism of the alkaline α-galactosidase AtAkαGal3. Schematic presentation of the proposed catalytic mechanism of the alkaline α-galactosidase AtAkαGal3 with raffinose as a donor and an acceptor. The main interactions of residues involved in substrate recognition and the catalytic process are shown: the nucleophile (Asp383), acid/base catalyst (Asp447), Lys381 and Arg443 for the −1 subsite, Trp78, Lys75, Asp446 and Tyr449 for the +1 subsite, Lys75, Trp77, Thr342 and Asp346 for sucrose at the secondary product-binding site, Asp346 for the +3 subsite and water molecules that help to stabilize the +2 subsite. (*a*) Raffinose as a donor substrate binds in the catalytic binding site. (*b*) After the glycosidic bond of raffinose has been cleaved, the sucrose product rotates 180° and binds to the secondary product-binding site. The sucrose is subsequently released and galactose remains in the catalytic pocket. (*c*) Another raffinose, which acts as an acceptor substrate, enters the pocket and moves towards the galactose that remains at the −1 subsite. (*d*) The acceptor raffinose is engaged with galactose, resulting in the production of stachyose.

The structure of D383A in complex with hydrolyzed raffinose reveals the products galactose at the −1 subsite and sucrose at the secondary binding site. Unexpectedly, after raffinose has been hydrolyzed between galactose and glucose at subsites −1 and +1, respectively, the sucrose shifts and rotates by 180°. The glucose moiety of sucrose in the +1 subsite is thus replaced by a fructose moiety and is bound in a different orientation and position to the related moieties of raffinose and stachyose in other D383A–substrate complexes (Fig. 8 ▸). We specify this newly discovered binding position as the secondary product-binding site. A 180° rotation of the sucrose product after the hydrolysis of raffinose is possible because the catalytic pocket is wide, which provides sufficient space for flipping and binding (Fig. 9 ▸). After rotation, Thr342 and Asp346 help to stabilize the glucose and fructose of the sucrose product, respectively. Furthermore, Lys75, which helps to stabilize the sugar moiety in the +1 subsite, also makes a strong interaction with the glucose of the sucrose product in the +3 subsite, indicating that the conserved residues Lys75 and Trp78 in the WW box region are important for stabilization of both the substrate and the product in the +1 subsite, according to the proposed mechanism in Fig. 10 ▸. Lys381 might be the key stabilizer during the catalytic process.

In summary, the alkaline α-galactosidase AtAkαGal3 from *A. thaliana* exhibits a dual function: it not only catalyzes the hydrolysis of α-d-galactose from galacto-oligosaccharides under alkaline conditions but can also synthesize stachyose using raffinose, instead of galactinol, as the galactose donor. Structural analyses of AtAkαGal3 and its D383A mutant in complex with various substrates and products, including galactose, galactinol, raffinose, stachyose and sucrose, elucidate four complete subsites (–1 to +3) and a new secondary product-binding site. The AtAkαGal3 structure in this study is the first representative structure of an alkaline α-galactosidase and may also serve a structural model for the raffinose family of RFO synthases.

## Supplementary Material

PDB reference: AtAkαGal3, wild type, complex with galactose, 7exf

PDB reference: D383A mutant, complex with galactose, 7exg

PDB reference: complex with galactinol, 7exh

PDB reference: complex with rafffinose, 7exj

PDB reference: complex with galactose and sucrose, 7exq

PDB reference: complex with stachyose, 7exr

Supplementary Figures. DOI: 10.1107/S2059798323000037/ji5027sup1.pdf

## Figures and Tables

**Figure 7:**
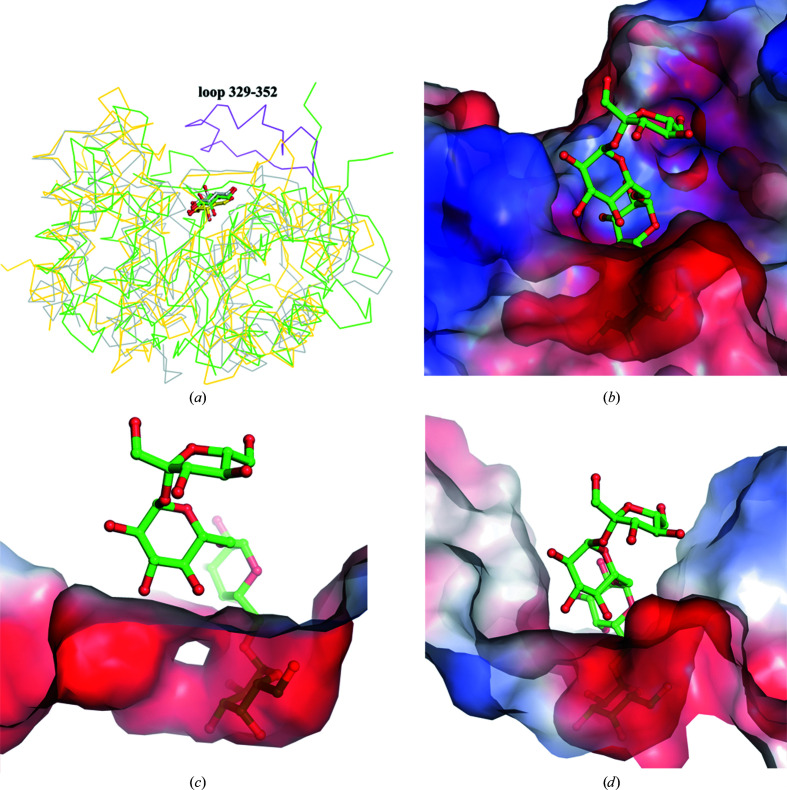
Superimposition of catalytic (α/β)_8_-barrel domains. (*a*) Superimposition of the catalytic (α/β)_8_-barrel domains of the alkaline α-galactosidase AtAkαGal3 (green), rice α-galactosidase (gray) and *T. maritima* α-galactosidase (yellow) with the product galactose at the respective −1 subsite. An extra loop (residues 329–352, magenta) exists exclusively in AtAkαGal3. (*b*) The complex of D383A with stachyose reveals that an extra loop helps to stabilize the product or stachyose substrate at the +2 and +3 subsites in the alkaline α-galactosidase AtAkαGal3. An electrostatic surface is shown (blue, positive charge; red, negative charge). (*c*, *d*) Docking of stachyose from the D383A complex into the structures of rice α-galactosidase (*c*) and *T. maritima* α-­galactosidase (*d*) shows that both catalytic binding sites are shallow such that they can accommodate only one galactose, compared with AtAkαGal3 that can hold four-sugar moieties such as stachyose.

**Table 1:** Data-collection and refinement statistics

	SeMet-AtAkαGal3							
	Peak	Inflection	AtAkαGal3–galactose	D383A–galactose	D383A–galactinol	D383A–rafffinose	D383A–stachyose	D383A–galactose–sucrose
PDB code			7exf	7exg	7exh	7exj	7exr	7exq
Data collection								
Beamline	TPS 05A		TLS 15A	TPS 05A	BL44XU	BL44XU	TPS 05A	TLS 15A
Space group	*P*2_1_2_1_2_1_	*P*2_1_2_1_2_1_	*P*2_1_2_1_2_1_	*P*2_1_2_1_2_1_	*P*2_1_2_1_2_1_	*P*2_1_2_1_2_1_	*P*2_1_2_1_2_1_	*P*2_1_2_1_2_1_
*a*, *b*, *c* (Å)	95.75, 96.00, 104.59	104.69, 183.76, 183.74	94.94, 103.64, 181.21	94.03, 103.74, 182.70	97.52, 103.65, 182.43	92.60, 103.30, 181.44	97.59, 103.75, 182.37	97.71, 104.10, 182.42
Wavelength (Å)	0.97918	0.97935	1.000	0.999	0.900	0.900	0.999	1.000
Resolution (Å)	30–3.10	30–3.50	30–2.17	30–2.05	30–2.63	30–2.47	30–2.0	30–2.2
No. of observed reflections	331082	245197	568408	714794	326607	370537	785125	500553
No. of unique reflections	35194	26054	95207	111655	105231	118865	124558	92281
Completeness (%)	98.6 (100)	99.0 (100)	98.2 (83.7)	99.3 (95.4)	98.6 (99.1)	98.5 (96.6)	99.9 (99.5)	97.2 (100)
Multiplicity	9.4 (9.7)	9.4 (9.8)	6.0 (6.0)	6.4 (5.8)	3.1 (3.2)	3.1 (3.3)	6.3 (5.4)	5.4 (5.6)
〈*I*/σ(*I*)〉	21.0 (3.3)	15.7 (4.4)	16.8 (2.2)	26.06 (3.3)	9.28 (2.3)	10.56 (1.8)	26.87 (2.4)	22.39 (2.6)
*R*_merge_ (%)	11.7 (78.0)	14.2 (63.0)	7.8 (77.6)	7.5 (55.4)	11.8 (66.2)	7.4 (60.1)	9.3 (59.7)	8.5 (78.9)
CC_1/2_			0.829	0.906	0.763	0.812	0.855	0.880
Refinement								
Resolution (Å)			30.0–2.17	30.0–2.05	30.0–2.63	30.0–2.47	30.0–2.00	30.0–2.20
*R*_work_/*R*_free_			0.21/0.26	0.20/0.25	0.17/0.23	0.19/0.24	0.18/0.21	0.19/0.25
No. of atoms								
Protein			11246	11220	11232	11240	11240	11226
Ligand			24	24	46	68	90	70
Water			177	579	52	16	544	416
*B* factors (Å^2^)								
Protein			61.3	31.1	53.5	71.6	42.6	40.4
Ligand			53.0	20.5	60.0	73.4	55.5	49.3
Water			44.2	31.8	41.0	56.2	44.8	38.0
R.m.s.d.								
Bond lengths (Å)			0.014	0.018	0.017	0.013	0.020	0.017
Bond angles (°)			1.728	2.015	1.948	1.733	2.125	1.936

**Table 2:** Structural alignment of the (α/β)_8_-barrel domain of AtAkαGal3 with rice α-galactosidase and *T. maritima* α-galactosidase and a sequence alignment between AtAkαGal3 and the raffinose/stachyose (Raf/Sta) synthases show the conserved, variable and potential hydrogen-bond interactions (distances within 3.5 Å) at the −1, +1, +2 and +3 subsites among these glycosidase families Bold labels indicate the key catalytic residues at the active site. Substrates and products: Gal, galactose; Gol, galactinol; Raf, raffinose; Sta, stachyose.

Substrate/product							
Gal	Gol	Raf	Sta	AtAkαGal3 residues 75–78, 201–531 (PDB entries 7exf, 7exg, 7exh, 7exj, 7exr, 7exq)	Rice α-galactosidase (GH27) residues 6–271 (PDB entry 1uas)	*T. maritima* α-galactosidase (GH36) residues 165–481 (PDB entry 6gvd)	Raf/Sta synthases (no structure, based on sequence)
			+3	Waters			Asp/Val
		+2	+2	Tyr449			Trp
				Asp447(w)			Asp
				Asp451(w)			Thr, (Arg)/Gln, (Arg, Glu)
	+1	+1	+1	Lys75			Lys
				Trp78		Trp65	Trp
				Asp446	Trp164 (−1)		Trp
							Asp/1E
−1	−1	−1	−1	Asp243	Asp51	Asp220	Asp
				Asp244	Asp52	Asp221	Asp
				Trp307	Lys128	Trp257	Trp
				Lys381		Lys325	Lys
				**Asp383**	**Asp130**	**Asp327**	Asp
				Arg443	Arg181	Arg383	Arg
				**Asp447**	**Asp185**	**Asp387**	Asp
