# Supplementary material for: Structural insight into the hydrolase and synthase activities of an alkaline α-galactosidase from Arabidopsis from complexes with substrate/product
Source: Acta Crystallogr D Struct Biol. 2023 Jan 20;79(Pt 2):154–67. doi: 10.1107/S2059798323000037 (PMC9912918; doi:10.1107/S2059798323000037)
Supplement: Supplementary file 1 [file d-79-00154-sup1.pdf]

STRUCTURAL
BIOLOGY

Volume 79 (2023)

Supporting information for article:

Structural insight into the hydrolase and synthase activities of an alkaline α-galactosidase from *Arabidopsis* from complexes with substrate/product

Phimonphan Chuankhayan, Ruey-Hua Lee, Hong-Hsiang Guan, Chein-Chih Lin, Nai-Chi Chen, Yen-Chieh Huang, Masato Yoshimura, Atsushi Nakagawa and Chun-Jung Chen

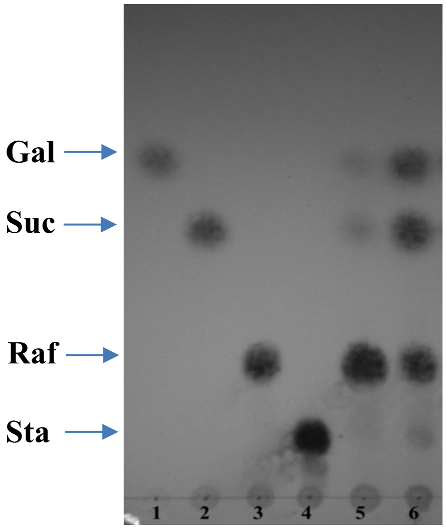

Figure S1 Thin-layer chromatography (TLC) analysis. Hydrolysis reaction of AtAkαGal3, compared with rice raffinose synthase, toward raffinose as a substrate. Lane 1: standard (std.) galactose (Gal); lane 2: std. sucrose (Suc); lane 3: std. raffinose (Raf); lane 4: std. stachyose (Sta); lane 5: raffinose incubated with rice raffinose synthase; lane 6: raffinose incubated with AtAkαGal3.

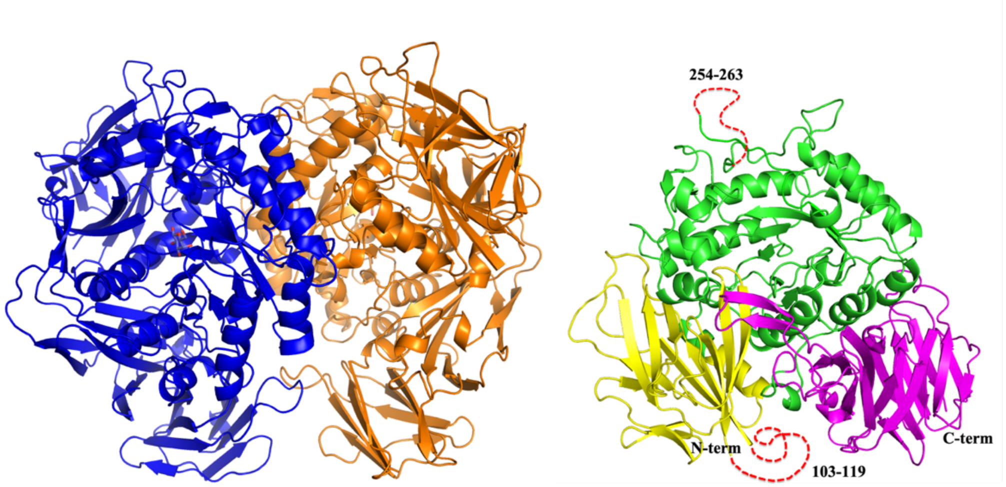

Figure S2 Crystal structure of AtAkαGal3. (A) Two AtAkαGal3 molecules (blue and orange) exist in one asymmetric unit. (B) The overall structure of a functional monomer is composed of three domains: the N-terminal domain (yellow), the catalytic domain (green) and the C-terminal domain (magenta). Disordered residues 103-119 and 254-263 without defined electron density are shown in red dashed lines.

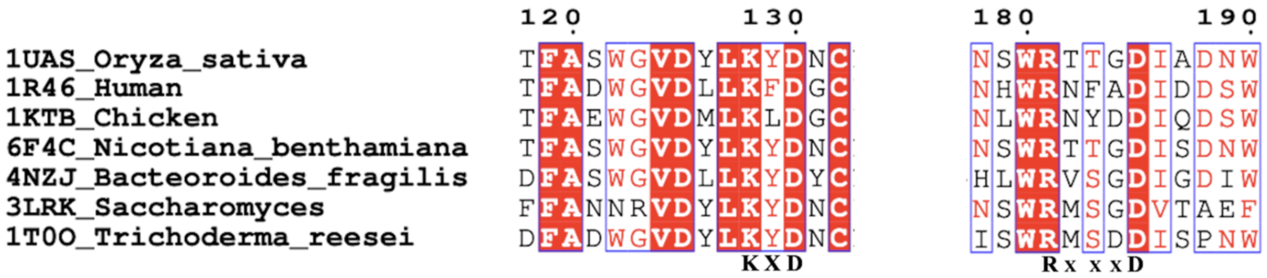

Figure S3 Alignment of the catalytic region of acidic a-galactosidases with known structures. PDB 1UAS: α-galactosidase from *Oryza sativa*, 1R46: human α-galactosidase, 1KTB: chicken α-N-acetylgalactosaminidase, 6F4C: α-galactosidase from *Nicotiana benthamiana*, 4NFJ: α-galactosidase from *Bacteoroides fragilis*, 3LRK: α-galactosidase from *Saccharomyces cerevisiae*, 1T0O: α-galactosidase from *Trichoderma reesei.*

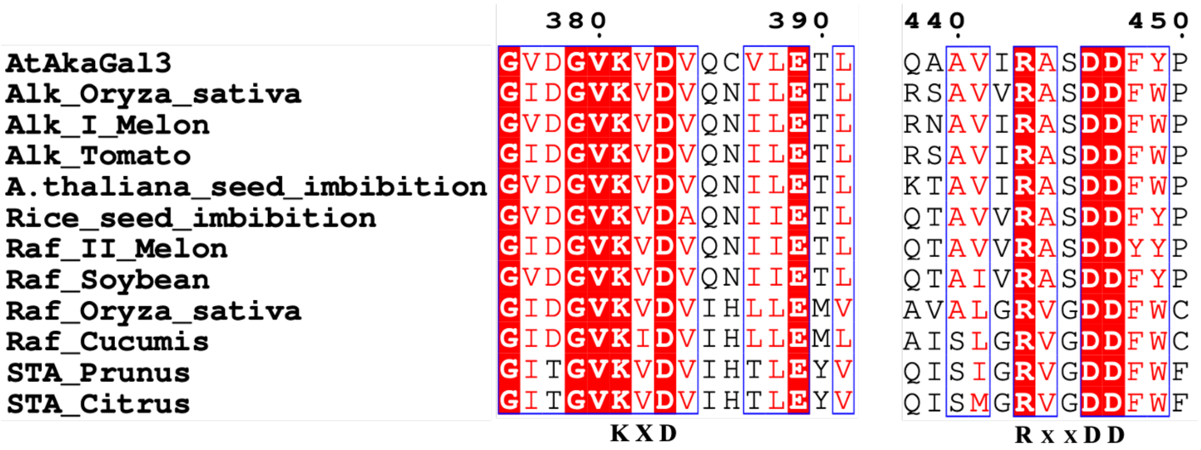

Figure S4 Alignment of the catalytic region of alkaline α-galactosidase and RFO synthases from different plants. There are AtAkαGal3, alkaline α-galactosidases from rice (AF251068) (Alk_Oryza_sativa), melon (AAM75139) (Alk_I_Melon), tomato (NP_001234763) (Alk_Tamato), and seed imbibition protein from AtAkαGal1 (Atlg55740) (A.thaliana_seed_imbibition), and rice (ABF99470) (Rice_seed_imbibition), RFO synthases from melon (NP_001284472) (Raf_II_Melon), soybean (XP_006576826) (Raf_Soybean), rice (XP_015621501) (Raf_Oryza_sativa), cucumber (AAD02832) (Raf_cucumis), yoshino cherry (PQQ02596) (STA_Prunus) and clementine (XP_006444535) (STA_Citrus), showed two conserved motifs of nucleophile: $_{381}$KVD$_{383}$ and $_{443}$RxxDD$_{447}$. The residue numbers are based on the AtAkαGal3 sequence.

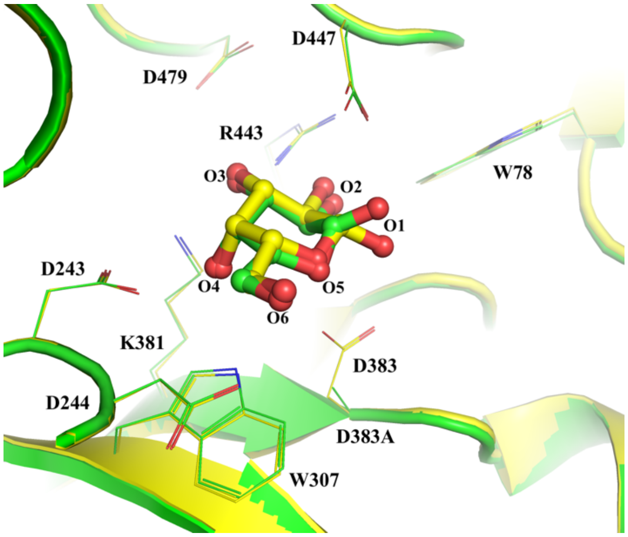

Figure S5 Structural comparison of the mutant D383 and wild-type in complex with galactose. Superimposed two galactose moieties of the D383A mutant (green stick) and wild-type AtAkαGal3 (yellow stick) show that the O1 hydroxyl group at the C1-carbon are in α- and β-anomeric forms, respectively.
